# Rethinking Machine-Learning Metrics in Schistosomiasis Control: Toward Predictive Equity, Field Readiness, and Biological Foresight

**DOI:** 10.4269/ajtmh.25-0470a

**Published:** 2025-09-30

**Authors:** Nathkapach Kaewpitoon Rattanapitoon, Phatsakul Thitimahatthanukusol, Thirayu Meererksom, Schawanya Kaewpitoon Rattanapitoon

**Affiliations:** Parasitic Disease Research FMC Medical Center Nakhon Ratchasima, Thailand E-mail: nathkapach.ratt@gmail.com; Faculty of Engineering and Technology Rajamangala University of Technology Isan Nakhon Ratchasima, Thailand E-mail: Phatsakul.th@rmuti.ac.th; Kasira House of Technology Nakhon Ratchasima, Thailand E-mail: thirayu.m@gmail.com; Parasitic Disease Research FMC Medical Center Nakhon Ratchasima, Thailand E-mail: Schawanya.ratt@g.sut.ac.th

Dear Editor,

We read with great interest the article by Chen et al., which presents a well-validated machine learning (ML) approach to predicting the prevalence of heavy-intensity schistosomiasis in sub-Saharan Africa.^1^ Their integration of environmental, demographic, and morbidity prevalence data represents a commendable advance toward achieving the WHO’s 2030 goals.

However, we wish to raise several considerations to enrich the scientific dialogue around ML’s application in parasitic disease control:

## Model accuracy is not field utility.

While the authors report impressive metrics (*R*^2^ > 0.80, MAE = 0.02), predictive accuracy alone does not guarantee epidemiological utility. High-performing models often face limitations in field interpretation, especially when nonlinear interactions are opaque to programmatic stakeholders. Tools such as the SHapley Additive exPlanations or Local Interpretable Model-Agnostic Explanations should be employed to dissect model predictions at local levels. These methods have been successfully used in ML-based helminth risk mapping in Southeast Asia and could be pivotal in enhancing trust and uptake by national programs.^2^

## Underrepresentation of biological complexity.

The model’s strong reliance on morbidity prevalence as the primary predictor may obscure crucial parasitological dynamics. For instance, schistosome–host–snail interactions are heterogeneous across microhabitats and seasons.^3^ The omission of snail distribution models and ecological niches may limit predictions, particularly in focal transmission zones.

Furthermore, recent studies in Senegal and northern Cameroon report hybridization between *S. haematobium* and *S. bovis*, altering pathogenesis and diagnostic sensitivity.^4^ Such evolutionary plasticity could confound intensity-based metrics unless species and strain identification is incorporated.

## Spatial resolution and demographic disaggregation.

Although the authors utilized georeferenced survey points from 12 countries, spatial heterogeneity within implementation units remains substantial. A study in Makenene, Cameroon demonstrated how schistosomiasis risk varied sharply within 5 km^2^.^5^ Incorporating hyperlocal WASH data and age–sex stratification could improve granularity, particularly where adult infections are underrepresented. Similarly, recent findings from Tanzania and Mali underscore how male occupational exposure and peri-urban informal settlements correlate with higher heavy-intensity burdens.^6^^,^^7^

## Toward predictive equity and field readiness.

Model generalizability is as vital as its precision. While the authors acknowledge the exclusion of *S. japonicum* and *S. intercalatum*, we propose that model architecture be adapted for modular inputs (including localized diagnostics, snail distribution, and behavioral risk indices), enabling transferability beyond current geographies. Notably, open-source platforms like *ESPEN Collect* and WHO’s *DHI Tracker* can provide modular integration channels.

We encourage the authors to explore ensemble geostatistical–ML models, combining Bayesian hierarchical frameworks^8^ with explainable ML to bridge ecological realism and data-driven forecasts.

## References

[1] Chen X Le J Hu Y, 2024. Predicting schistosomiasis intensity in Africa: A machine learning approach to evaluate the progress of WHO roadmap 2030. Am J Trop Med Hyg 111: 73–79. 38772355 10.4269/ajtmh.23-0751 PMC11229641

[2] Luo C Wang Y Su Q Zhu J Tang S Bergquist R Zhang Z Hu Y, 2022. Mapping schistosomiasis risk in Southeast Asia: A systematic review and geospatial analysis. Int J Epidemiol 52: 1137–1149. 10.1093/ije/dyac227 36478466

[3] Jones IJ, , 2021. Schistosome infection in Senegal: Ecological drivers for *Schistosoma haematobium* and *S. mansoni*. PLoS Negl Trop Dis 15: e0009712. 34570777 10.1371/journal.pntd.0009712 PMC8476036

[4] Catalano S Sène M Diouf ND, , 2020. Hybrid schistosomes: An emerging threat. Trends Parasitol 36: 899–911.

[5] Mewamba EM, , 2022. Fine-scale mapping of *Schistosoma mansoni* infections and infection intensities in sub-districts of Makenene in the Centre region of Cameroon. PLoS Negl Trop Dis 16: e0010852. 36227962 10.1371/journal.pntd.0010852 PMC9595529

[6] Mazigo HD Nuwaha F Dunne DW Kaatano GM Angelo T Kepha S Kinung’hi SM, 2017. *Schistosoma mansoni* infection and its related morbidity among adults living in selected villages of Mara Region, north-western Tanzania: A cross-sectional exploratory study. Korean J Parasitol 55: 533–540. 29103268 10.3347/kjp.2017.55.5.533 PMC5678472

[7] Sacko M Magnussen P Deita AD Traoré S Landouré A Doucouré A Madsen H Vennervald BJ, 2011. Impact of *Schistosoma haematobium* infection on urinary tract pathology, nutritional status and anaemia in school-aged children in two different endemic areas of the Niger River Basin, Mali. Acta Trop 120 *(**Suppl 1**):* S142–S150. 21195046 10.1016/j.actatropica.2010.12.009

[8] Clements AC Moyeed R Brooker S, 2006. Bayesian geostatistical prediction of the intensity of infection with *Schistosoma mansoni* in East Africa. Parasitology 133: 711–719. 16953953 10.1017/S0031182006001181 PMC1783909

